# Open Versus Hybrid and Total Minimally Invasive Transthoracic Ivor Lewis Esophagectomy Following Neoadjuvant FLOT Chemotherapy: An Australian and New Zealand Cohort Study

**DOI:** 10.1002/wjs.70391

**Published:** 2026-05-04

**Authors:** Brendan Desmond, Maneesha De Silva, Darren J. Wong, David I. Watson, Cuong P. Duong, Tim Bright, Ahmad Aly, Margaret Lee, Kevin Chan, Garett Smith, David L. Chan, Neil Merrett, Sivakumar Gananadha, Yick Ho Lam, Harsh Kanhere, Mark Smithers, Michael Bozin, Matthew Read, Krinal Mori, Mary‐Ann Johnson, Enoch Wong, Sarah A. Martin, Geraldine Ooi, Yahya Al‐Habbal, Chon Hann Liew, Robert Bohmer, Jurstine Daruwalla, Mo Ballal, Rukshan Ranjan, Andrew D. MacCormick, Sharon Pattison, Nicholas Evennett, Jason Robertson, James Tan, Alexandra Gordon, Simon Bann, David S. Liu, SPACE‐FLOT ANZ Investigators

**Affiliations:** ^1^ Upper Gastrointestinal Surgery Unit, Division of Surgery, Anaesthesia and Procedural Medicine Austin Hospital Heidelberg Victoria Australia; ^2^ Department of Gastroenterology, Anaesthesia and Procedural Medicine Austin Hospital Heidelberg Victoria Australia; ^3^ College of Medicine and Public Health Flinders University Adelaide South Australia Australia; ^4^ Department of Surgery Flinders Medical Centre Adelaide South Australia Australia; ^5^ Division of Cancer Surgery Peter MacCallum Cancer Centre Melbourne Victoria Australia; ^6^ Division of Cancer Research Peter MacCallum Cancer Centre Melbourne Victoria Australia; ^7^ Department of Medical Oncology Box Hill Hospital Melbourne Victoria Australia; ^8^ Department of General Surgery, Upper Gastrointestinal Unit Royal Brisbane and Women's Hospital Brisbane Queensland Australia; ^9^ Upper Gastrointestinal Surgery Unit Royal Northshore Hospital Sydney New South Wales Australia; ^10^ Northern Clinical School, Faculty of Medicine and Health University of Sydney Sydney New South Wales Australia; ^11^ Upper Gastrointestinal Surgical Unit Bankstown‐Lidcombe Hospital Bankstown New South Wales Australia; ^12^ School of Medicine Western Sydney University Campbelltown New South Wales Australia; ^13^ Upper Gastrointestinal Surgery Unit Canberra Hospital Canberra Australia; ^14^ Upper Gastrointestinal Surgery Unit Lyell McEwin Hospital Adelaide South Australia Australia; ^15^ Upper Gastrointestinal Surgery Unit Royal Adelaide Hospital Adelaide South Australia Australia; ^16^ Upper Gastrointestinal Surgery Unit Princess Alexandra Hospital Brisbane Queensland Australia; ^17^ Upper Gastrointestinal Surgery Unit St Vincent's Hospital Melbourne Victoria Australia; ^18^ Upper Gastrointestinal Surgery Unit Northern Hospital Melbourne Victoria Australia; ^19^ Upper Gastrointestinal Surgery Unit Box Hill Hospital Melbourne Victoria Australia; ^20^ Upper Gastrointestinal Surgery Unit Monash Medical Centre Melbourne Victoria Australia; ^21^ Department of Surgery Monash University Melbourne Victoria Australia; ^22^ Upper Gastrointestinal Surgery Unit Western Health Melbourne Victoria Australia; ^23^ Upper Gastrointestinal Surgery Unit Bendigo Health Bendigo Victoria Australia; ^24^ Upper Gastrointestinal Surgery Unit Royal Hobart Hospital Hobart Tasmania Australia; ^25^ Upper Gastrointestinal Surgery Unit Launceston General Hospital Launceston Tasmania Australia; ^26^ Upper Gastrointestinal Surgery Unit Fiona Standley Hospital Perth Western Australia Australia; ^27^ Upper Gastrointestinal Surgery Unit Christchurch Hospital Christchurch New Zealand; ^28^ Upper Gastrointestinal Surgery Unit Middlemore Hospital Auckland New Zealand; ^29^ Department of Surgery University of Auckland Auckland New Zealand; ^30^ Southern Blood and Cancer Service Health New Zealand Te Whatu Ora—Southern Dunedin New Zealand; ^31^ Department of Pathology Otago Medical School—Dunedin Campus, University of Otago Ōtākou Whakaihu Waka Dunedin New Zealand; ^32^ Upper Gastrointestinal Surgery Unit Auckland City Hospital Auckland New Zealand; ^33^ Upper Gastrointestinal Surgery Unit North Shore Hospital Takapuna New Zealand; ^34^ Upper Gastrointestinal Surgery Unit Palmerston North Hospital Palmerston North New Zealand; ^35^ Upper Gastrointestinal Surgery Unit Wellington Regional Hospital Newtown New Zealand; ^36^ Victorian Interventional Research and Trials Unit The University of Melbourne Department of Surgery, Austin Health Heidelberg Victoria Australia

**Keywords:** esophageal cancer, minimally invasive esophagectomy, textbook outcomes

## Abstract

**Background:**

In Australian and Aotearoa New Zealand (ANZ), it is unclear whether minimally invasive transthoracic Ivor Lewis esophagectomy (MIO) is superior to open techniques with regards to perioperative and oncological outcomes. Most evidence on this topic have been derived from high‐volume centers prior to the advent of perioperative FLOT chemotherapy. How these findings are applicable to the ANZ context, where oesophagectomies are typically performed in low‐moderate volume centers, is unknown. This study compares perioperative outcomes and long‐term survival between patients undergoing transthoracic open versus hybrid and total MIO following neoadjuvant FLOT chemotherapy across multiple ANZ centers.

**Methods:**

Retrospective analysis of transthoracic oesophagectomies undertaken between 2017 and 2022 following neoadjuvant FLOT chemotherapy from 22 ANZ centers. The primary endpoint was the rate of major (Clavien‐Dindo grade ≥ 3) postoperative complication. Secondary endpoints included nodal yield, surgery time, length‐of‐stay, and rates of perioperative complications, positive resection margins, ICU readmissions, in‐hospital mortality, 30‐day hospital readmissions, textbook outcome, adjuvant chemotherapy delivered, as well as disease free (DFS) and overall survival (OS).

**Results:**

Open esophagectomy, hybrid MIO and total MIO was performed in 155 (62.5%), 61 (24.6%), and 32 (12.9%) patients, respectively. From open to total MIO, there was a stepwise decrease in the rate of major postoperative complications (Open: 38.7%, hybrid MIO: 29.5%, total MIO: 15.6%, *p* = 0.032). This was associated with reduced length‐of‐stay [Median(IQR), Open: 14 (11–23), hybrid MIO: 13 (11–23), total MIO: 10 (8–12), *p* = 0.031], and lower rates of pulmonary (Open: 49.0%, hybrid MIO: 42.6%, total MIO: 28.1%, *p* = 0.031), cardiac (Open: 20.0%, hybrid MIO: 6.6%, total MIO: 3.1%, *p* = 0.006), sepsis (Open: 18.7%, hybrid MIO: 8.2%, total MIO: 3.1%, *p* = 0.022), and wound (Open: 12.3%, hybrid MIO: 3.3%, total MIO: 0.0%, *p* = 0.019) complications. No significant differences were observed in other perioperative endpoints. Moreover, institutional factors including enhanced recovery after surgery programs and hospital case volume interacted with surgical technique to influence postoperative complication rates. Importantly, adjusted DFS and OS were comparable between the three groups.

**Conclusions:**

In ANZ, MIO was associated with fewer complications and comparable survival compared to open transthoracic esophagectomy. These findings support the safety of MIO in lower‐volume settings in the era of perioperative FLOT chemotherapy.

## Graphical abstract

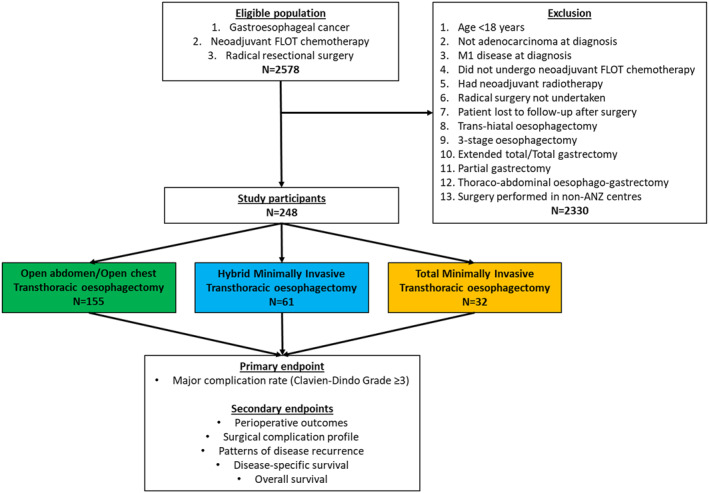

## 1 Introduction

Esophagectomy remains the cornerstone of curative‐intent treatment for locally advanced esophageal and gastroesophageal junction cancers. The Ivor Lewis approach, a two‐field transthoracic esophagectomy, is widely practiced [1]. Traditionally performed via laparotomy and thoracotomy, this procedure has increasingly incorporated minimally invasive techniques, including hybrid (either component performed minimally invasively) and total (both components performed minimally invasively) minimally invasive esophagectomy (MIO) [2].

Early landmark European trials have supported this evolution. The TIME trial reported significantly fewer pulmonary complications with total MIO compared to open surgery [3]. Similarly, the MIRO trial found that hybrid MIO resulted in lower rates of intraoperative and postoperative complications, particularly pulmonary morbidity [4]. Furthermore, the ROBOT trial, comparing robot assisted total MIO versus open surgery found significantly decreased rates of major postoperative complications in those who underwent MIO [5]. Moreover, registry data from the United Kingdom has suggested superior oncological outcomes with minimally invasive techniques [6]. However, the recently reported ROMIO trial, the largest and most comprehensive of the four randomized studies, demonstrated equivalent outcomes between hybrid MIO and open approaches across perioperative and functional endpoints [7], thus restoring clinical equipoise to the question of whether minimally invasive approaches (total or hybrid) to transthoracic esophagectomy confer any perioperative and oncological advantages over open surgery.

It is important to highlight that whilst these trials are informative, they represent the experience from high‐volume European centers with centralized upper gastrointestinal cancer services. However, the Australian and Aotearoa New Zealand (ANZ) healthcare context differs significantly, with limited centralization of gastroesophageal cancer services and comparatively lower procedural volumes per center than those in European trials [8]. This contextual difference is particularly relevant given the established learning curve associated with MIO. The technical complexities of minimally invasive approaches raise key questions about whether the potential benefits demonstrated in high‐volume European centers translate to the ANZ and other healthcare settings around the world, where centralization of esophagectomy services has not occurred [8].

The recently published SPACE‐FLOT study presents a valuable opportunity to examine these questions in a contemporary ANZ cohort [9]. This cohort is especially relevant as all patients received neoadjuvant FLOT (5‐fluorouracil, leucovorin, oxaliplatin, and docetaxel) chemotherapy, which may now be considered the dominant standard of care based on the ESOPEC trial showing superior survival outcomes compared to neoadjuvant chemoradiation [10].

Accordingly, this study aims to compare open versus hybrid and total minimally invasive transthoracic esophagectomy undertaken in ANZ centers following neoadjuvant FLOT chemotherapy with regard to perioperative and oncological endpoints.

## 2 Methods

### 2.1 Study Design

**FIGURE 1:**
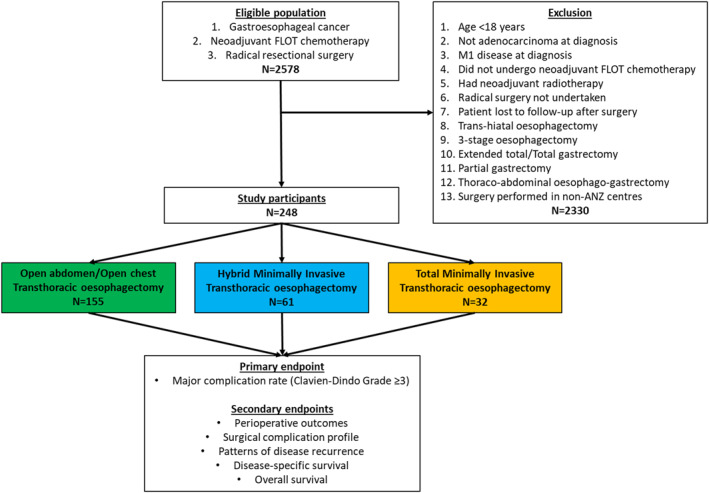
Study flow chart.

This study analyzed all patients within the SPACE‐FLOT registry who received neoadjuvant FLOT chemotherapy and underwent elective transthoracic (Ivor Lewis) esophagectomy for gastroesophageal adenocarcinoma between 1 January 2017 to 1 January 2022 across 22 ANZ hospitals (Supporting Information S1: Table S1). Open esophagectomy was defined as surgery where both a laparotomy and thoracotomy were performed. Total MIO was defined as surgery in which there was no thoracotomy or laparotomy performed. Hybrid MIO was defined as thoracotomy with laparoscopy consistent with MIRO and ROMIO trials [4, 7]. Surgical approach was determined by established unit practice patterns rather than case‐by‐case selection. All patients underwent workup per ESMO guidelines including pre‐operative nutritional assessment [11]. Institutional ERAS and prehabilitation data were collected and analyzed. The inclusion and exclusion criteria for this study are shown in Figure 1. This study was registered with the Australian New Zealand Clinical Trials Registry (ACTRN12622000180718) and was approved by the Peter MacCallum Cancer Center Human Research Ethics Committee (HREC/76492/PMCC) and all sites.

### 2.2 Data Collection and Quality Assurance

An online research electronic data capture (REDCap) database (tested and validated by the Peter MacCallum Data Systems, Research Computing Facility) was used to collect data. Interobserver variations were minimized by utilizing training sessions for data collectors, in‐program prompting, and real‐time data entry support. Two investigators independently cleaned the data. A mean (standard deviation) accuracy rate of 97.8% (2.3) was demonstrated with random auditing of 10% of data fields from all sites.

### 2.3 Study Outcomes and Definitions

The primary endpoint of this study was the rate of major postoperative complication defined by the Clavien‐Dindo classification system as grade ≥ 3 [12]. Secondary endpoints included duration of surgery, pathological margin status, total nodes examined, length of stay (LOS), intensive care readmissions, in‐hospital mortality, 30‐day hospital readmissions, perioperative outcomes achieving textbook outcome criteria [13], individual surgical complications as defined by the Esophagectomy Complications Consensus Group (ECCG) [14], proportion of patients receiving adjuvant treatment, pattern of tumor recurrence, disease free (DFS) and overall survival (OS).

Esophagectomy achieving textbook outcome criteria was defined per internationally validated consensus criteria by Kalff et al. [13] including nine quality metrics: (1) tumor‐negative resection margins (R0), (2) no intraoperative complications, (3) ≥ 20 lymph nodes retrieved and examined, (4) no major postoperative complications, (5) no anastomotic leakage including all grades defined by the Esophagectomy Complications Consensus Group (ECCG) [14], (6) no intensive care unit (ICU) readmission, (7) no length of stay (LOS) ≥ 14 days, (8) no in‐hospital mortality, and (9) no readmission related to the surgical procedure. DFS was determined from the date of surgery to the date of disease recurrence based on clinical, endoscopic and/or radiological examinations. OS was determined from the date of surgery to the date of death from any cause. Those alive at study termination were censored at the time of last contact.

Baseline comorbidities were quantified using the Charlson Comorbidity Index, which also estimates the probability of survival at 10 years [15]. Clinicians' assessment of each patient's overall surgical fitness was classified using the American Society of Anesthesiologists (ASA) score [16]. Postoperative medical complications were defined according to the European Perioperative Clinical Outcome definitions [17]. The severity of surgical complications was classified using the Clavien‐Dindo system [12]. Tumor location was categorized according to Siewert's classification [18]. Pathological tumor response to neoadjuvant FLOT chemotherapy was trichotomized into complete, partial and minimal response according to the SPACE‐FLOT study [9]. All tumors were staged using the Eighth Edition AJCC Cancer Staging Manual [19].

### 2.4 Power Calculation

Based on the TIME, MIRO and ROBOT [3, 4, 5] trials demonstrating a 20%–28% difference in major postoperative complication rate between open and MIO groups, we powered this study to detect a 20% absolute difference in major postoperative complication rate between open versus hybrid MIO and open versus total MIO groups (alpha 0.05, 80% power). Given the anticipated ratio of 5:2:1 for open versus hybrid MIO versus total MIO cases [9], the calculated sample size required was 240 patients (open: 150, hybrid MIO: 60, total MIO: 30) for this study.

### 2.5 Statistical Analysis

Univariate comparisons between study groups were performed using Fisher's exact test and Student's t test for categorical and continuous variables respectively. For non‐parametric continuous data, the Mann‐Whitney *U* test was used. Where comparisons involved more than two variables, the chi‐square test was applied for categorical data and the analysis of variance test with repeated measures was used for continuous data. Statistical analyses were conducted by co‐author biostatistician DJW. Unadjusted DFS and OS were analyzed using log rank test. Multivariate Cox proportional hazards models were fitted adjusting for age, sex, body mass index, Charlson Comorbidity Index, smoking status, ASA score, tumor location, completion of neoadjuvant FLOT chemotherapy, tumor grade, presence of lymphovascular infiltration and perineural invasion, pathological tumor stage, tumor regression grading, and receipt of adjuvant chemotherapy. The proportional hazards assumption was tested for all models. Two‐tail *p* values < 0.050 and odds (OR) or hazards (HR) ratio with 95% confidence intervals that did not cross one were considered statistically significant. Statistical analyses were undertaken using Prism version 10.2.2 (GraphPad Software, San Diego, CA, USA) and R version 4.1.0 (R Foundation for Statistical Computing, Vienna, Austria).

## 3 Results

### 3.1 Baseline Characteristics

In total, 248 patients were evaluated. Of these, 155 (62.5%) were open oesophagectomies, 61 (24.6%) were hybrid MIOs, and 32 (12.9%) were total MIOs (Figure 1). Their mean age was 62.8 (SD 8.8) years, and 216 (87.1%) patients were male. A total of 232 patients (93.5%) had locally advanced (cT ≥ 2 and/or cN1+) cancers, with 223 (90.0%) cases classified as distal esophageal/Siewert 1 and 2 tumors and 25 (10.0%) located in the gastric cardia/Siewert 3. Overall, 225 patients (90.7%) had completed all 4 cycles of neoadjuvant FLOT. The median follow‐up duration was 24.6 (IQR 14.6–40.2) months post‐surgery. Baseline demographic, neoadjuvant treatment, and clinicopathological tumor characteristics were comparable between study groups (Table 1).

**TABLE 1:** Baseline characteristics.

Characteristics	Open esophagectomy (*n* = 155)	Hybrid MIO (*n* = 61)	Total MIO (*n* = 32)	*p* value
Demographics				
Age (years), mean (SD)	63.1 (8.4)	63.0 (9.1)	60.8 (9.9)	0.393
Sex (male), *n* (%)	135 (87.1)	50 (82.0)	31 (96.9)	0.126
BMI (kg/m^2^), mean (SD)	28.8 (5.4)	27.6 (5.3)	26.9 (3.3)	0.108
Charlson co‐morbidity index				0.203
Index score, median (IQR)	2 (1–3)	2 (2–3)	2 (1–3)	
Predicted 10‐year survival (%), mean (SD)	80.3 (21.8)	78.3 (22.9)	86.6 (16.5)	
Smoking status at time of surgery, *n* (%)				0.228
Active	20 (12.9)	7 (11.5)	0 (0.0)	
Former	76 (49.0)	37 (60.7)	20 (62.5)	
Never	56 (36.1)	17 (27.9)	11 (34.4)	
Unknown	3 (1.9)	0 (0.0)	1 (3.1)	
ASA grade at time of surgery, median (IQR)	2 (2–3)	3 (2–3)	2 (2–3)	0.050
Neoadjuvant treatment				0.113
Completed 4 cycles of FLOT, *n* (%)	140 (90.3)	53 (86.9)	32 (100.0)	
FLOT cycles completed, median (IQR)	4 (4–4)	4 (4–4)	4 (4–4)	
Clinical tumor features				
Anatomical location of tumor, *n* (%)				0.534
Distal esophageal/siewert 1 and siewert 2	137 (88.4)	57 (93.4)	29 (90.6)	
Siewert 3	18 (11.6)	4 (6.6)	3 (9.4)	
Clinical stage, *n* (%)				0.647
cT1 N0 M0	12 (7.7)	2 (3.3)	2 (6.3)	
cT2‐3 N0 M0	69 (44.5)	35 (57.4)	13 (40.6)	
cT4a N0 M0	2 (1.3)	0 (0.0)	0 (0.0)	
cT4b N0 M0	1 (0.6)	0 (0.0)	0 (0.0)	
cT1 N1 M0	3 (1.9)	0 (0.0)	2 (6.3)	
cT2‐3 N1 M0	62 (40.0)	23 (37.7)	15 (46.9)	
cT4a N1 M0	5 (3.2)	1 (1.6)	0 (0.0)	
cT4b N1 M0	1 (0.6)	0 (0.0)	0 (0.0)	
Tumor histology				
Histological diagnosis, *n* (%)				0.216
Adenocarcinoma	150 (96.8)	61 (100.0)	61 (100.0)	
Adenocarcinoma with squamous differentiation	5 (3.2)	0 (0.0)	0 (0.0)	
Tumor grade, *n* (%)				0.357
Well differentiated	8 (5.2)	5 (8.2)	1 (3.1)	
Moderately differentiated	52 (33.5)	20 (32.8)	5 (15.6)	
Poorly differentiated	75 (48.4)	25 (41.0)	18 (56.3)	
Undifferentiated	1 (0.6)	0 (0.0)	0 (0.0)	
Indeterminant	19 (12.3)	11 (18.0)	8 (25.0)	
Lymphovascular invasion, *n* (%)	61 (39.4)	18 (29.5)	10 (31.3)	0.335
Perineural invasion, *n* (%)	34 (21.9)	9 (14.8)	7 (21.9)	0.480
Tumor regression grading, *n* (%)				0.989
Complete responders	27 (17.4)	12 (19.7)	6 (18.8)	
Partial responders	91 (58.7)	34 (57.4)	19 (59.4)	
Minimal responders	37 (23.9)	14 (23.0)	7 (21.9)	
Pathological stage, *n* (%)				0.247
0	27 (17.4)	12 (19.7)	6 (18.8)	
1a	9 (5.8)	1 (1.6)	1 (3.1)	
1b	10 (6.5)	3 (4.9)	1 (3.1)	
1c	8 (5.2)	4 (6.6)	1 (3.1)	
2a	8 (5.2)	2 (3.3)	5 (15.6)	
2b	33 (21.3)	11 (18.0)	9 (28.1)	
3a	5 (3.2)	7 (11.5)	1 (3.1)	
3b	37 (23.9)	18 (29.5)	6 (18.8)	
4a	18 (11.6)	3 (4.9)	2 (6.3)	

Abbreviations: ASA, American Society of Anesthesiologist, BMI, Body mass index, IQR, Interquartile range, MIO, Minimally invasive esophagectomy, SD, Standard deviation.

### 3.2 Major Postoperative Complications

**FIGURE 2:**
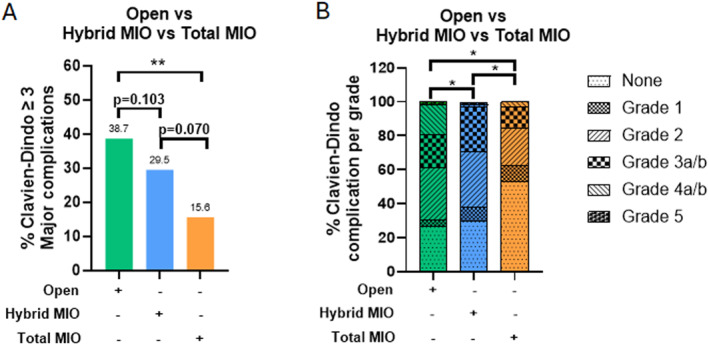
(A) Rate of major (Clavien‐Dindo grade ≥ 3) post‐operative complication and (B) overall complication profile, in patients who underwent open (open abdomen/open chest), hybrid (laparoscopic abdomen/open chest or open abdomen/thorascopic chest) or totally (laparoscopic abdomen and thoracoscopic chest) minimally invasive esophagectomy (MIO). *p < 0.05, **p < 0.01. Values above each column in (A) corresponds to actual major post‐operative complication rate.

Compared to open esophagectomy, total MIO was associated with a significantly lower rate of major postoperative complications (OR 0.29, 95% CI 0.12–0.75, *p* = 0.006). Despite not reaching statistical significance, there was a stepwise decrease in major postoperative complication rate between hybrid MIO versus open esophagectomy, and total MIO versus hybrid MIO (Figure 2A). Furthermore, the complication severity profile as classified by the Clavien‐Dindo system was significantly different between open, hybrid and total MIO groups (Figure 2B), with a higher proportion of grade 1 and 2 complications within the minimally invasive cohorts than the open group.

### 3.3 Factors Influencing Major Postoperative Complications

**FIGURE 3:**
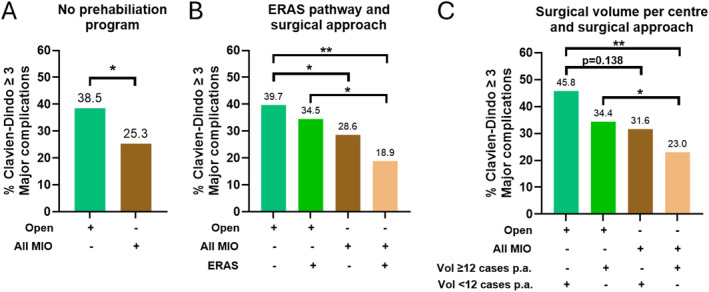
Rate of major (Clavien‐Dindo grade ≥ 3) post‐operative complication in patients who underwent open or any minimally invasive esophagectomy (MIO) in centers without prehabilitation (A), with or without an enhanced recovery after surgery pathway (ERAS) (B), and performing ≥ 12 or < 12 cases per center number per annum (p.a.) (C). *p < 0.05, **p < 0.01. Values above each column corresponds to actual major post‐operative complication rate.

As institutional factors such as prehabilitation [20], enhanced recovery after surgery (ERAS) programs [21], as well as hospital case volume [8, 22], may confound the impact of minimally invasive surgery, we conducted subgroup analyses excluding patients from centers who delivered prehabilitation, and assessed the effects of ERAS programs (present or absent) and esophagectomy case volume (≥ 12 or < 12 cases per center per year based on previous ANZ studies) [8], on the risk of developing a major postoperative complication. We found that in patients who did not undergo prehabilitation, MIO was associated with a significantly lower rate of major postoperative complication than open esophagectomy (Figure 3A). Furthermore, regardless of an ERAS program, patients who underwent MIO had a significantly lower rate of major postoperative complication than those who received an open esophagectomy (Figure 3B). Importantly, the combination of an ERAS program and MIO synergically lowered the rate of major postoperative complication (Figure 3B). Moreover, regardless of case volume, MIO was associated with a lower rate of major postoperative complication than open esophagectomy (Figure 3C). Notably, in centers who performed ≥ 12 oesophagectomies per year, MIO resulted in the lowest rate of major postoperative complication (Figure 3C).

### 3.4 Surgical Complication Profile

Compared to open esophagectomy, both hybrid and total MIO were associated with significantly lower rates of cardiac complications, arrhythmias requiring intervention, generalized sepsis, and surgical site infections (Table 2). Additionally, a significantly lower rate of pulmonary complications was found in the total MIO group when compared with those who underwent an open esophagectomy (Table 2). There was no significant difference in the incidence of individual postoperative complications between the hybrid and total MIO groups (Supporting Information S1: Table S2).

**TABLE 2:** Surgical complication profile according to Clavien‐Dindo and ECCG definitions.

Complications	Open esophagectomy (*n* = 155)	Hybrid MIO (*n* = 61)	Total MIO (*n* = 32)	Open versus hybrid		Open versus total	
				Or (95% CI)	*p* value	Or (95% CI)	*p* value
Clavien‐Dindo complication grade, *n* (%)				—	0.048	—	0.020
No complications	41 (26.5)	18 (29.5)	17 (53.1)				
Grade 1	6 (3.9)	5 (8.2)	3 (9.4)				
Grade 2	48 (31.0)	20 (32.8)	7 (21.9)				
Grade 3 a/b	30 (19.4)	16 (26.2)	4 (12.5)				
Grade 4 a/b	27 (17.4)	1 (1.6)	1 (3.1)				
Grade 5	3 (1.9)	1 (1.6)	0 (0.0)				
Pulmonary, *n* (%)							
All pulmonary complications	76 (49.0)	26 (42.6)	9 (28.1)	0.77 (0.44–1.40)	0.396	0.41 (0.17–0.92)	0.031
Pneumonia	58 (37.4)	20 (32.8)	7 (21.9)	0.86 (0.45–1.54)	0.523	0.47 (0.18–1.15)	0.093
Pleural effusion requiring drainage	34 (21.9)	9 (14.8)	3 (9.4)	0.62 (0.27–1.38)	0.234	0.37 (0.11–1.16)	0.104
Respiratory failure requiring intubation	14 (9.0)	5 (8.2)	2 (6.3)	0.90 (0.34–2.50)	0.845	0.67 (0.15–2.81)	0.609
Pneumothorax requiring intervention	6 (3.9)	1 (1.6)	1 (3.1)	0.41 (0.04–2.62)	0.404	0.80 (0.07–5.24)	0.840
Acute respiratory distress syndrome	7 (4.5)	2 (3.3)	1 (3.1)	0.72 (0.15–3.38)	0.682	0.68 (0.06–4.06)	0.723
Acute aspiration	5 (3.2)	2 (3.3)	0 (0.0)	1.0 (0.20–4.94)	0.984	—	0.303
Atelectasis requiring bronchoscopy	7 (4.5)	0 (0.0)	0 (0.0)	—	0.092	—	0.221
Tracheobronchial injury	3 (1.9)	0 (0.0)	0 (0.0)	—	0.274	—	0.428
Air leak > 10 days post‐surgery	5 (3.2)	0 (0.0)	0 (0.0)	—	0.156	—	0.303
Cardiac, *n* (%)							
All cardiac complications	31 (20.0)	4 (6.6)	1 (3.1)	0.28 (0.10–0.78)	0.016	0.13 (0.01–0.75)	0.021
Arrhythmias requiring intervention	29 (18.7)	4 (6.6)	1 (3.1)	0.30 (0.11–0.86)	0.025	0.14 (0.01–0.82)	0.029
CCF Requiring intervention	6 (3.9)	1 (1.6)	0 (0.0)	0.41 (0.04–2.62)	0.404	—	0.258
Myocardial infarction	2 (1.3)	0 (0.0)	0 (0.0)	—	0.373	—	0.518
Cardiac arrest requiring intervention	0 (0.0)	0 (0.0)	0 (0.0)	—	—	—	—
Pericarditis requiring treatment	0 (0.0)	0 (0.0)	0 (0.0)	—	—	—	—
Gastrointestinal, *n* (%)							
Anastomotic leak	31 (20.0)	11 (18.0)	4 (12.5)	0.88 (0.39–1.87)	0.742	0.57 (0.20–1.73)	0.322
Conduit necrosis	2 (1.3)	3 (4.9)	0 (0.0)	4.00 (0.79–22.57)	0.111	—	0.518
Ileus delaying enteral feeding	13 (8.4)	1 (1.6)	2 (6.3)	0.18 (0.02–1.19)	0.070	0.73 (0.16–3.12)	0.685
Small bowel obstruction	3 (1.9)	1 (1.6)	0 (0.0)	0.84 (0.06–5.76)	0.885	—	0.428
Chyle leak	7 (4.5)	3 (4.9)	0 (0.0)	1.09 (0.30–4.13)	0.899	—	0.221
Pancreatitis	0 (0.0)	0 (0.0)	0 (0.0)	—	—	—	—
Liver dysfunction	8 (5.2)	0 (0.0)	0 (0.0)	—	0.071	—	0.189
Acute diaphragmatic hernia	0 (0.0)	1 (1.6)	0 (0.0)	—	0.110	—	—
Infection, *n* (%)							
General sepsis	29 (18.7)	5 (8.2)	1 (3.1)	0.39 (0.16–1.00)	0.050	0.14 (0.01–0.82)	0.029
SSI requiring intervention	19 (12.3)	2 (3.3)	0 (0.0)	0.24 (0.05–1.00)	0.045	—	0.037
Clostridium difficile infection	1 (0.6)	1 (1.6)	0 (0.0)	2.57 (0.13–49.03)	0.492	—	0.649
Line infection requiring intervention	4 (2.6)	1 (1.6)	0 (0.0)	0.63 (0.05–3.92)	0.679	—	0.358
Intrathoracic abscess	18 (11.6)	4 (6.6)	2 (6.3)	0.53 (0.19–1.51)	0.269	0.51 (0.11–1.99)	0.372
Intraabdominal abscess	5 (3.2)	1 (1.6)	0 (0.0)	0.50 (0.04–3.78)	0.523	—	0.303
Neurological, *n* (%)							
Delirium	19 (12.3)	6 (9.8)	1 (3.1)	0.78 (0.30–2.00)	0.616	0.23 (0.02–1.44)	0.128
Cerebrovascular accident	0 (0.0)	0 (0.0)	0 (0.0)	—	—	—	—
Hematological, *n* (%)							
Bleeding requiring intervention	8 (5.2)	0 (0.0)	0 (0.0)	—	0.071	—	0.189
Venous thromboembolism	12 (7.7)	2 (3.3)	3 (9.4)	0.40 (0.09–1.69)	0.230	1.23 (0.35–4.33)	0.757
Urological, *n* (%)							
Acute kidney injury	10 (6.5)	1 (1.6)	0 (0.0)	0.24 (0.02–1.47)	0.148	—	0.140
Urinary tract infection	3 (1.9)	3 (4.9)	1 (3.1)	2.62 (0.60–11.42)	0.230	1.63 (0.12–11.23)	0.672
Skin, *n* (%)							
Wound dehiscence	7 (4.5)	0 (0.0)	0 (0.0)	—	0.092	—	0.221
Acute abdominal wall hernia	0 (0.0)	1 (1.6)	0 (0.0)	—	0.110	—	—

*Note:* Please refer to Supporting Information S1: Table S2 for Hybrid versus Open MIO comparisons.

Abbreviations: CCF, Congestive cardiac failure; CI, confidence interval; ECCG, Esophagectomy Complication Consensus Guidelines; MIO, Minimally invasive esophagectomy; OR, Odds ratio; SSI, Surgical site infection.

### 3.5 Perioperative Outcomes

Consistent with the above findings, there was a stepwise decrease in postoperative length of stay between hybrid MIO versus open esophagectomy [Median (IQR) days, 13 (11–23) versus 14 (11–23), *p* = 0.240], and total MIO versus open esophagectomy [Median (IQR) days, 10 (8–12) versus 14 (11–23), *p* = 0.045] (Table 3). Total MIO was also associated with a lower 30‐day hospital readmission rate when compared with hybrid MIO (OR 0.25, 95% CI 0.05–1.00, *p* = 0.030, Supporting Information S1: Table S3) and open esophagectomy (OR 0.29, 95% CI 0.07–1.18, *p* = 0.084, Table 3). There were no significant differences between the three groups with regards to other perioperative endpoints (Table 3 and Supporting Information S1: Table S3).

**TABLE 3:** Perioperative outcomes for esophagectomy.

Perioperative outcomes	Open esophagectomy (*n* = 155)	Hybrid MIO (*n* = 61)	Total MIO (*n* = 32)	Open versus hybrid		Open versus total	
				Or (95% CI)	*p* value	Or (95% CI)	*p* value
Duration of surgery, min, mean (SD)	451.9 (97.1)	465.8 (106.2)	448.4 (119.4)	—	0.643	—	0.983
Intraoperative complications, *n* (%)	13 (8.4)	3 (4.9)	1 (3.1)	0.57 (0.17–1.82)	0.381	0.35 (0.03–2.04)	0.303
R1 resection margins achieved, *n* (%)	14 (9.0)	1 (1.6)	2 (6.3)	0.16 (0.02–1.07)	0.054	0.67 (0.15–2.81)	0.609
Lymph node yield, median (IQR)	23 (15–30)	19 (15–26)	20 (17–26)	—	0.162	—	0.704
Intensive care readmission, *n* (%)	29 (18.7)	8 (13.1)	3 (9.4)	0.66 (0.29–1.47)	0.326	0.45 (0.14–1.44)	0.202
In‐hospital mortality, *n* (%)	3 (1.9)	1 (1.6)	0 (0.0)	0.84 (0.06–5.76)	0.885	—	0.428
30‐day hospital readmission, *n* (%)	29 (18.7)	13 (21.3)	2 (6.3)	1.18 (0.59–2.42)	0.664	0.29 (0.07–1.18)	0.084
Length of stay, days, median (IQR)	14 (11–23)	13 (11–23)	10 (8–12)	—	0.240	—	0.045
Textbook outcome achieved, *n* (%)	27 (17.4)	11 (18.0)	9 (28.1)	1.04 (0.47–2.29)	0.915	1.86 (0.77–4.25)	0.162
Start adjuvant chemotherapy, *n* (%)	104 (67.1)	42 (68.9)	26 (81.3)	1.08 (0.56–2.02)	0.804	2.12 (0.81–5.28)	0.113

Abbreviations: CI, confidence interval; IQR, Interquartile range; OR, Odds ratio; SD, Standard deviation.

### 3.6 Achieving Textbook Outcome Criteria

Overall, textbook outcome criteria were achieved in 17.4% of open cases, 18.0% of hybrid MIO cases, and 28.1% of total MIO cases. This was not significantly different between the three groups (Supporting Information S1: Table S4). In total, only 137 (55.2%) patients achieved a lymph node yield of ≥ 20. Although not statistically significant, the median nodal yield was higher in the open group [Median (IQR) count, open 23 (15–30) versus hybrid MIO 19 (15–26) versus total MIO 20 (17–26), *p* = 0.175]. Lymph node yield ≥ 20 was seen in 58.7% of open, 49.2% of hybrid, and 50.0% of total MIO cases, respectively (Supporting Information S1: Table S4).

### 3.7 Oncological Outcomes

**FIGURE 4:**
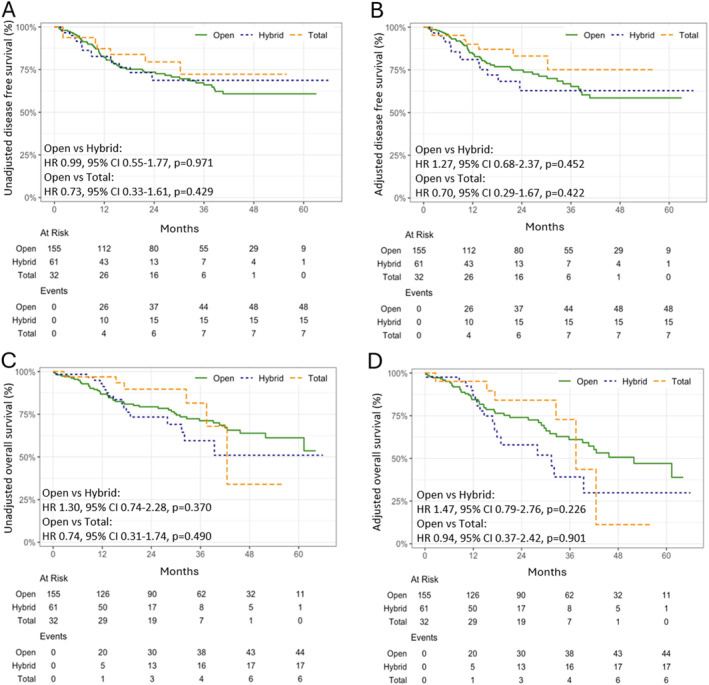
Unadjusted and adjusted disease‐free survival (A, B) and unadjusted and adjusted overall survival (C, D) comparing patients who underwent open versus hybrid or totally minimally invasive esophagectomy (MIO).

Unadjusted and adjusted DFS and OS was not statistically different between patients who underwent open versus hybrid or total MIO (Figure 4A–D). Similarly, there were no significant differences between these three groups with respect to their patterns of disease recurrence (Supporting Information S1: Figure S1–S3).

## 4 Discussion

This study is the first multicentre real‐world analysis of esophagectomy outcomes in the FLOT era across ANZ. We found that MIO, particularly total MIO, was associated with fewer major postoperative complications and shorter hospital stays. There were no significant differences in meeting textbook outcome criteria and survival between open, hybrid and total MIO groups.

Consistent with the TIME and ROBOT trials [3, 5], we identified significantly fewer pulmonary complications and shorter LOS in patients who underwent a total MIO than an open esophagectomy. Moreover, our open group had higher rates of cardiac arrhythmias, sepsis, and wound complications compared to both hybrid and total MIO groups. These findings align with data from the MIRO trial, which demonstrated reduced postoperative complications with hybrid transthoracic esophagectomy [4]. It is important to note that the TIME and ROBOT trials compared open versus total MIO and showed greater reductions in pulmonary complication rates than those reported in the MIRO trial, which compared open versus hybrid approaches. In this context, our analysis which examined major (Open 38.7%, hybrid MIO 29.5%, total MIO 15.6%) and pulmonary complications (Open 49.0%, hybrid MIO 42.6%, total MIO 28.1%) and demonstrated that total MIO is superior to hybrid MIO and open surgery, is in keeping with the relative findings of MIRO, TIME and ROBOT trials [3, 4, 5].

Our overall finding of lower major postoperative complication rate in the MIO group compared to the open group, accounting for the effects of prehabilitation, ERAS programs and center volume, demonstrate that MIO offers enhanced perioperative outcomes in the ANZ context despite lower institutional volumes. Notably, the lowest rates of major complications were observed when MIO was combined with ERAS and higher case volumes, indicating that major complications are clearly influenced by these institutional factors. This is consistent with recent ANZ data showing that higher hospital volume is associated with reduced postoperative mortality and morbidity following esophagectomy [8, 22]. Taken together, this evidence suggests that centralization of esophagectomy services across ANZ may have benefits through increasing institutional volume, but also facilitating the implementation of ERAS, prehabilitation, and other facets of perioperative care, which can synergistically enhance patient outcomes.

From a health economic perspective, our findings of reduced major complications (total MIO 15.6% vs. open 38.7%) and shorter length of stay (median 10 vs. 14 days) have important cost implications. Recent Australian data demonstrate that each increase in Clavien‐Dindo severity grade adds approximately USD $13,594 to hospital costs, with major complications doubling admission costs [23]. This further underscores the potential clinical and economic benefit of minimally invasive techniques in esophagectomy.

The overall rate of meeting textbook outcome criteria in this study was lower than international benchmarks, with European studies reporting rates as high as 45% [24]. By comparison, textbook outcome criteria was achieved in 17% of open, 18% of hybrid, and 28% of total MIO cases in this study. The performance of our cohort in the criteria of lymph node yield > 20 and anastomotic leak rate (Supporting Information S1: Table S4) was inferior to other studies and contributed to the decreased rate of achieving textbook outcome [24].

A lymph node yield ≥ 20 was achieved in only 58.7% of open cases and 49.5% of MIO cases. In comparison, median nodal yields of 30 with MIO have been reported internationally, and higher nodal harvests (> 30 nodes) have been associated with improved oncological outcomes [25, 26]. This discrepancy between the ANZ and international experience may reflect differences in case volume. Indeed, we observed that even within this ANZ cohort, Centers performing ≥ 12 oesophagectomies per year achieved higher lymph node yields (Supporting Information S1: Figure S4) and lower complication rates for both open and MIO, but particularly for MIO (Figure 3C). These volume‐outcome relationships align with recent ANZ data [8, 22] and provide evidence for centralization. Concentrating cases in high‐volume centers would optimize surgical expertise in MIO, facilitate ERAS implementation, enable specialized pathological processing, and likely improve outcomes.

The superior oncological outcomes associated with minimally invasive esophagectomy in previous studies were not replicated in our cohort [7]. The absence of a survival benefit in the total and hybrid MIO groups may also be due to the use of FLOT chemotherapy, which may potentially mitigate the detrimental effects of a relatively lower lymph node yield in both open and MIO groups [27]. It is possible that systemic therapy may compensate for limitations in surgical quality. Additionally, the increased complication rates observed in the open group may counterbalance potential survival benefits related to higher nodal yield, given that postoperative morbidity is associated with inferior oncological outcomes [28, 29].

Despite the strengths of a large, contemporary dataset with uniform neoadjuvant therapy, rigorous data quality control measures, and statistical adjustments we acknowledge several limitations. The non‐randomized design introduces potential selection bias, particularly as patients with complex surgical histories or anatomy may have been preferentially assigned to open approaches. However, the absence of clinically meaningful differences in baseline characteristics between groups (Table 1) suggests that major systematic bias was limited, and reflects the use of unit‐based rather than case‐by‐case allocation of surgical approach across ANZ centers.

Because this was a multicentre real‐world study, there were no prospectively defined, uniform criteria for assigning individual patients to open, hybrid, or total minimally invasive esophagectomy. Instead, surgical approach was determined by unit‐level practice shaped by local expertise, training, and infrastructure. Some residual selection bias therefore cannot be excluded. Importantly, however, this study was designed to evaluate the effectiveness and safety of different esophagectomy strategies as they are actually delivered within a heterogeneous, non‐centralized healthcare system, rather than to define prescriptive patient‐level indications for one operative technique over another. The consistency of outcomes across centers, volumes, and perioperative pathways supports the internal validity of these findings, while recognizing that they should not be interpreted as mandating a single approach for all patients or surgeons.

Additionally, the SPACE‐FLOT registry does not differentiate between conventional laparoscopic and robotic surgery. Nonetheless, this study offers valuable insights into real‐world practice across ANZ, where geographical and logistical constraints limit the centralization of gastroesophageal cancer care. In this context, the demonstrated feasibility and oncological safety of minimally invasive esophagectomy in lower‐volume settings has relevance for other non‐centralized systems, while underscoring the need for continued quality improvement (e.g., lymphadenectomy, pathological processing and perioperative care) to meet international standards.

## 5 Conclusions

In this multicentre ANZ cohort, in the era of perioperative FLOT chemotherapy, MIO was associated with fewer major complications than open surgery. Furthermore, MIO was non‐inferior to open surgery regarding meeting textbook outcome criteria and cancer‐specific survival. Our findings demonstrate MIO feasibility and safety in ANZ while underscoring need for service centralization. Volume‐outcome relationships for complications and lymph node yield provide evidence‐based rationale for policy reform to meet international benchmarks. These data support the feasibility and safety of MIO in contemporary ANZ practice and highlights the importance for these procedures to be delivered in centralized services across the two nations.

## Author Contributions

**Brendan Desmond:** investigation, writing – original draft, methodology, visualization, writing – review and editing. **Maneesha De Silva:** investigation, writing – original draft, methodology, visualization, writing – review and editing. **Darren J. Wong:** conceptualization, investigation, writing – original draft, methodology, visualization, writing – review and editing, software, formal analysis. **David I. Watson:** investigation, funding acquisition, writing – original draft, writing – review and editing, methodology, project administration. **Cuong P. Duong:** investigation, funding acquisition, writing – review and editing, methodology, project administration, supervision. **Tim Bright:** investigation, funding acquisition, writing – review and editing, methodology, formal analysis. **Ahmad Aly:** investigation, funding acquisition, writing – review and editing, methodology, formal analysis, supervision. **Margaret Lee:** investigation, funding acquisition, writing – review and editing, methodology, formal analysis, supervision. **Kevin Chan:** investigation, funding acquisition, writing – review and editing, methodology, data curation, supervision. **Garett Smith:** investigation, writing – review and editing, methodology, data curation, supervision. **David L. Chan:** investigation, methodology, writing – review and editing, data curation, supervision. **Neil Merrett:** investigation, methodology, writing – review and editing, data curation, supervision. **Sivakumar Gananadha:** investigation, writing – review and editing, methodology, data curation, supervision. **Yick Ho Lam:** investigation, methodology, writing – review and editing, data curation, supervision. **Harsh Kanhere:** investigation, methodology, writing – review and editing, data curation, supervision. **Mark Smithers:** investigation, methodology, writing – review and editing, data curation, supervision. **Michael Bozin:** investigation, methodology, writing – review and editing, data curation, supervision. **Matthew Read:** investigation, methodology, writing – review and editing, data curation, supervision. **Krinal Mori:** investigation, methodology, writing – review and editing, data curation, supervision. **Mary‐Ann Johnson:** investigation, methodology, writing – review and editing, data curation, supervision. **Enoch Wong:** investigation, methodology, writing – review and editing, data curation, supervision. **Sarah A. Martin:** methodology, investigation, writing – review and editing, data curation, supervision. **Geraldine Ooi:** investigation, methodology, writing – review and editing, data curation, supervision. **Yahya Al‐Habbal:** investigation, methodology, writing – review and editing, data curation, supervision. **Chon Hann Liew:** investigation, methodology, writing – review and editing, data curation, supervision. **Robert Bohmer:** investigation, methodology, writing – review and editing, data curation, supervision. **Jurstine Daruwalla:** investigation, methodology, writing – review and editing, supervision, data curation. **Mo Ballal:** investigation, methodology, writing – review and editing, data curation, supervision. **Rukshan Ranjan:** investigation, methodology, writing – review and editing, data curation, supervision. **Andrew D. MacCormick:** investigation, methodology, writing – review and editing, data curation, supervision. **Sharon Pattison:** investigation, methodology, writing – review and editing, data curation, supervision. **Nicholas Evennett:** investigation, methodology, writing – review and editing, data curation, supervision. **Jason Robertson:** investigation, methodology, writing – review and editing, data curation, supervision. **James Tan:** investigation, methodology, writing – review and editing, data curation, supervision. **Alexandra Gordon:** investigation, methodology, writing – review and editing, data curation, supervision. **Simon Bann:** investigation, methodology, writing – review and editing, supervision, data curation. **David S. Liu:** conceptualization, investigation, funding acquisition, writing – original draft, writing – review and editing, visualization, validation, methodology, software, formal analysis, project administration, resources, supervision.

## Funding

The study was supported by the North Eastern Melbourne Integrated Cancer Service, Peter MacCallum Cancer Foundation, Austin Medical Research Foundation, Royal Australasian College of Surgeons, Australian Gastro‐Intestinal Trials Group, Cancer Council Victoria, and the Victorian Cancer Agency.

## Ethics Statement

This study was registered with the Australian New Zealand Clinical Trials Registry (ACTRN12622000180718) and was approved by the Peter MacCallum Cancer Center Human Research Ethics Committee (HREC/76492/PMCC).

## Conflicts of Interest

The authors declare no conflicts of interest.

## Supporting information


Supporting Information S1

## Data Availability

The data that support the findings of this study are available on request from the corresponding author. The data are not publicly available due to privacy or ethical restrictions.
