# Supplementary material for: Open Versus Hybrid and Total Minimally Invasive Transthoracic Ivor Lewis Esophagectomy Following Neoadjuvant FLOT Chemotherapy: An Australian and New Zealand Cohort Study
Source: World J Surg. 2026 May 4;50(6):1650–62. doi: 10.1002/wjs.70391 (PMC13242059; doi:10.1002/wjs.70391)
Supplement: Supplementary file 1 — Supporting Information S1 [file WJS-50-1650-s001.docx]

**SUPPLEMENTARY MATERIAL**

**Open versus hybrid and total minimally invasive transthoracic Ivor Lewis oesophagectomy following neoadjuvant FLOT chemotherapy: An Australian and New Zealand Cohort Study**

**Authors**

Brendan Desmond (*MMedSci, FRACS*),^1^ Maneesha De Silva (MD),^1^ Darren J. Wong (*PhD, FRACP*),^5^ David I. Watson (*PhD, FRACS*),^6,7^ Cuong P. Duong (*PhD, FRACS*),^2,3^ Tim Bright (*MS, FRACS*),^6,7^ Ahmad Aly (*MS, FRACS*),^1^ Margaret Lee (MBBS, FRACP),^8^ Kevin Chan (*MBBS, FRACS*),^9^ Garett Smith (*MS, FRACS*),^10^ David L. Chan (*PhD, FRACP*),^10,31^ Neil Merrett (*PhD, FRACS*),^11,32^ Sivakumar Gananadha (*MS, FRAC*S),^12^ Yick Ho Lam (*MBBS, FRACS*),^13^ Harsh Kanhere (*MS, FRACS*),^14^ Mark Smithers (*PhD, FRACS*),^15^ Michael Bozin (*PhD, FRACS*),^2^ Matthew Read (*PhD, FRACS*),^16^ Krinal Mori (*MS, FRACS*),^17^ Mary-Ann Johnson (*MBBS, FRACS*),^34^ Enoch Wong (*MSurg, FRACS*),^34^ Sarah A. Martin (*FRACS, FACS*),^18,33^ Geraldine Ooi (*PhD, FRACS*),^18,33^ Yahya Al-Habbal (*FRACS, FACS*),^19^ Chon Hann Liew (*MBBS, FRACS*),^20^ Robert Bohmer (*MBChB, FRACS*),^21^ Jurstine Daruwalla (*PhD, FRACS*),^22^ Mo Ballal (*MPhil, FRACS*),^23^ Rukshan Ranjan (*MBChB, FRACS*),^24^ Andrew D. MacCormick (*PhD, FRACS*),^25,36^ Sharon Pattison (*PhD, FRACP*),^26,35^ Nicholas Evennett (*MD, FRACS*),^27^ Jason Robertson (*MMedSc, FRACS*),^28^ James Tan (*MBChB, FRACS*),^28^ Alexandra Gordon (*MBChB, FRACS*),^29^ Simon Bann (*MD, FRACS*),^30^ SPACE-FLOT ANZ Investigators,^37^ David S. Liu (*PhD, FRACS*)^1,2,3,4^

**Affiliations**

1. Upper Gastrointestinal Surgery Unit, Division of Surgery, Anaesthesia and Procedural Medicine, Austin Hospital, 145 Studley Road, Heidelberg, Victoria, 3084, Australia
2. Division of Cancer Surgery, Peter MacCallum Cancer Centre, 305 Grattan Street, Melbourne, Victoria, 3000, Australia
3. Division of Cancer Research, Peter MacCallum Cancer Centre, 305 Grattan Street, Melbourne, Victoria, 3000, Australia
4. Victorian Interventional Research and Trials Unit, The University of Melbourne Department of Surgery, Austin Health, 145 Studley Road, Heidelberg, Victoria, 3084, Australia.
5. Department of Gastroenterology, Anaesthesia and Procedural Medicine, Austin Hospital, 145 Studley Road, Heidelberg, Victoria, 3084, Australia
6. College of Medicine and Public Health, Flinders University, South Australia, Australia.
7. Department of Surgery, Flinders Medical Centre, South Australia, Australia.
8. Department of Medical Oncology, Box Hill Hospital, Victoria, Australia
9. Department of General Surgery, Upper Gastrointestinal Unit, Royal Brisbane and Women’s Hospital, Brisbane, Australia
10. Upper Gastrointestinal Surgery Unit, Royal Northshore Hospital, New South Wales, Australia
11. Upper Gastrointestinal Surgical Unit, Bankstown-Lidcombe Hospital, New South Wales, Australia
12. Upper Gastrointestinal Surgery Unit, Canberra Hospital, Canberra, Australia
13. Upper Gastrointestinal Surgery Unit, Lyell McEwin Hospital, South Australia, Australia
14. Upper Gastrointestinal Surgery Unit, Royal Adelaide Hospital, South Australia, Australia
15. Upper Gastrointestinal Surgery Unit, Princess Alexandra Hospital, Brisbane, Australia
16. Upper Gastrointestinal Surgery Unit, St Vincent’s Hospital, Victoria, Australia
17. Upper Gastrointestinal Surgery Unit, Northern Hospital, Victoria, Australia
18. Upper Gastrointestinal Surgery Unit, Monash Medical Centre, Victoria, Australia
19. Upper Gastrointestinal Surgery Unit, Western Health, Victoria, Australia
20. Upper Gastrointestinal Surgery Unit, Bendigo Health, Victoria, Australia
21. Upper Gastrointestinal Surgery Unit, Royal Hobart Hospital, Tasmania, Australia
22. Upper Gastrointestinal Surgery Unit, Launceston General Hospital, Tasmania, Australia
23. Upper Gastrointestinal Surgery Unit, Fiona Standley Hospital, Western Australia, Australia
24. Upper Gastrointestinal Surgery Unit, Christchurch Hospital, Aotearoa New Zealand
25. Upper Gastrointestinal Surgery Unit, Middlemore Hospital, Aotearoa New Zealand
26. Southern Blood and Cancer Service, Health New Zealand Te Whatu Ora – Southern, Aotearoa New Zealand
27. Upper Gastrointestinal Surgery Unit, Auckland City Hospital, Aotearoa New Zealand
28. Upper Gastrointestinal Surgery Unit, North Shore Hospital, Aotearoa New Zealand
29. Upper Gastrointestinal Surgery Unit, Palmerston North Hospital, Aotearoa New Zealand
30. Upper Gastrointestinal Surgery Unit, Wellington Regional Hospital, Aotearoa New Zealand
31. Northern Clinical School, Faculty of Medicine and Health, University of Sydney, New South Wales, Australia
32. School of Medicine, Western Sydney University, Campbelltown, New South Wales, Australia
33. Department of Surgery, Monash University, Victoria, Australia
34. Upper Gastrointestinal Surgery Unit, Box Hill Hospital, Victoria, Australia
35. Department of Pathology, Otago Medical School – Dunedin Campus, University of Otago Ōtākou Whakaihu Waka, Aotearoa New Zealand
36. Department of Surgery, University of Auckland, Aotearoa New Zealand
37. See Appendix 1. Collaborators

**Corresponding author**

A/Prof. David S Liu

*MBBS (Hons), BMedSc, PhD, FRACS, FAANZGOSA*

Division of Cancer Surgery, Peter MacCallum Cancer Centre, 305 Grattan Street, Melbourne, Victoria, 3052, Australia

Upper Gastrointestinal Surgery Unit, Division of Surgery, Anaesthesia and Procedural Medicine, Austin Hospital, 145 Studley Road, Heidelberg, Victoria, 3084, Australia

Victorian Interventional Research and Trials Unit, Department of Surgery, University of Melbourne, Austin Precinct, 145 Studley Road, Heidelberg, Victoria, 3084, Australia

Phone: +613 8559 5000

Email: liu.davidsh@gmail.com

ORCID ID: 0000-0001-8936-4123

Twitter/X: @Dr.DavidSLiu

**TABLE OF CONTENTS**

**Supplementary Appendixes**

Appendix 1. Collaborators 4

Appendix 2. Collaborators’ contribution 5

**Supplementary Results**

Table S1. Participating sites 6

Table S2. Surgical complication profile according to Clavien-Dindo and ECCG definitions 7

Table S3. Perioperative outcomes for oesophagectomy 8

Table S4. Textbook outcomes for oesophagectomy 9

Figure S1. 10

Figure S2 11

Figure S3 12

Figure S4 13

**Appendix 1. Collaborators**

David S. Liu**,** Brendan Desmond, Maneesha De Silva, Darren J. Wong, David I. Watson**,** Cuong P. Duong, Tim Bright, Ahmad Aly, Margaret M. Lee, Katheryn Hall, Sonia Gill, Chao Cheng, Su Kah Goh, Matthew Read, James Tan, Sean Stevens, Enoch Wong, Geraldine Ooi, Yick Ho Lam**,** Eunice Lee**,** David Williams, Louise Jackett**,** Kevin Chan, Garett Smith, David L. Chan, Neil Merrett, Sivakumar Gananadha, Harsh Kanhere, Lauren Kennedy, Mark Smithers, Janine Thomas, Michael Bozin, Lynn Chong, Krinal Mori, Mary-Ann Johnson, Sarah A. Martin, Geraldine Ooi, Val Usatoff, Rod Jacobs, Yahya Al-Habbal, Chon Hann Liew, Fredrick Huynh, Robert Bohmer, Girish Pande, Jurstine Daruwalla, Mo Ballal, Deanna Lee, Rukshan Ranjan, Andrew D. MacCormick, James Wilkins, Sharon Pattison, Nicholas Evennett, Jason Robertson, Mark Pang, Alexandra Gordon, Simon Bann, Yu Kai Lim, Aleksandra Edmundson, Joe Q. Wei, Aldenb Lorenzo, Sam Alhayo, Aaditya Narendra, Aadil Rahim, Rocita Ho, Jeremy Granger, Steven Tran, Michalis Koullouros, Alain Nguyen, Christina McVeay, Siang Wei Gan, Eve Hopping, Iain Thomson, Andrew Barbour, David Gotley, Adam Frankel, Riteshkumar Patel, Shaun Jin Hui Chew, Kevin Lah, Sonia Gill, Stephen A. Barnett, Vijayaragavan Muralidharan, Samantha Phillips, Wael Jamel, Bung-Kook Ko, Shantanu Joglekar, Ashray Rajagopalan, Joseph Jaya, Yat Cheung Chung, Saania Peeroo, Marek Bak, Jonathan Tiong, Zhei Zhou, Amy Crowe, Ryan Newbold, Bethanie Trainor, Mei Lynn Pac Soo, Vaibhavee Khandelwal, Nicholas Eikelboom, Kyungchul Kim, Emily Moran, Joshua Hammerschlag, Brendan Desmond, Joel D'Souza, Jacky Lu, Rachel McLay-Barnes, Alexandra Gower, Jenny Choi, Yu Kai Lim, Douglas Wood, Kate Whytock

**Appendix 2. Collaborator’s contribution**

**Coordinating principal investigator:** David S. Liu

**Manuscript writing committee:** Brendan Desmond, Maneesha De Silva, Darren J. Wong, David S. Liu

**Study steering committee:** David S. Liu, Cuong P. Duong, David I. Watson, Tim Bright, Ahmad Aly, Margaret M. Lee

**Study management group:** David S. Liu, Katheryn Hall, Sonia Gill, Chao Cheng, Su Kah Goh, Darren J. Wong, Tim Bright, Cuong P. Duong, David I. Watson, Matthew Read, James Tan, Sean Stevens, Enoch Wong, Geraldine Ooi, Yick Ho Lam

**Study coordinator:** Katheryn Hall

**Biostatistician:** Darren J. Wong

**Database technicians:** Su Kah Goh, Eunice Lee

**Gastrointestinal pathologists:** David Williams, Louise Jackett

**New Zealand study lead:** James Tan

**Hospital leads**

(Royal Brisbane and Women’s Hospital, Australia) Kevin Chan, (Royal Northshore Hospital, Australia) Garett Smith, David L. Chan, (Bankstown Hospital, Australia) Neil Merrett, (Canberra Hospital, Australia) Sivakumar Gananadha, (Flinders Medical Centre, Australia) Tim Bright, David I. Watson, (Lyell McEwin Hospital, Australia) Yick Ho Lam, (Royal Adelaide Hospital, Australia) Harsh Kanhere, Lauren Kennedy, (Princess Alexandra Hospital, Australia) Mark Smithers, Janine Thomas, (Peter MacCallum Cancer Centre, Australia) Cuong Duong, Michael Bozin, (Austin Hospital, Australia) Ahmad Aly, David S. Liu, (St Vincent’s Hospital, Australia) Matthew Read, Lynn Chong, (Northern Hospital, Australia) Krinal Mori, (Box Hill Hospital, Australia) Mary-Ann Johnson, Enoch Wong, Margaret M. Lee, (Monash Medical Centre, Australia) Sarah A. Martin, Geraldine Ooi, (Western Hospital, Australia) Val Usatoff, Rod Jacobs, Yahya Al-Habbal, (Bendigo Hospital, Australia) Chon Hann Liew, Fredrick Huynh, (Royal Hobart Hospital, Australia) Robert Bohmer, (Launceston General Hospital, Australia) Girish Pande, Jurstine Daruwalla, (Fiona Stanley Hospital, Australia) Mo Ballal, Deanna Lee, Enoch Wong, (Christchurch Hospital, New Zealand) Rukshan Ranjan, (Middlemore Hospital, New Zealand) Andrew D. MacCormick, James Wilkins, (Health New Zealand Te Whatu Ora – Southern, New Zealand) Sharon Pattison, (Auckland City Hospital, New Zealand) Nicholas Evennett, James Wilkins, (North Shore Hospital, New Zealand) Jason Robertson, Mark Pang, James Tan, (Palmerston North Hospital, New Zealand) Alexandra Gordon, (Wellington Regional Hospital, New Zealand) Simon Bann, Yu Kai Lim

**Investigators**

(Royal Brisbane and Women’s Hospital, Australia) Aleksandra Edmundson, (Royal Northshore Hospital, Australia) Joe Q. Wei, (Bankstown Hospital, Australia) Aldenb Lorenzo, Sam Alhayo, Aaditya Narendra, (Canberra Hospital, Australia) Aadil Rahim, (Flinders Medical Centre, Australia) Yick Ho Lam, Rocita Ho, Jeremy Granger, Steven Tran, Michalis Koullouros, (Lyell McEwin Hospital, Australia) Alain Nguyen, Christina McVeay, (Royal Adelaide Hospital, Australia) Siang Wei Gan, Eve Hopping, (Princess Alexandra Hospital, Australia) Iain Thomson, Andrew Barbour, David Gotley, Adam Frankel, (Peter MacCallum Cancer Centre, Australia) Riteshkumar Patel, Shaun Jin Hui Chew, (Austin Hospital, Australia) Kevin Lah, Sonia Gill, Stephen A. Barnett, Vijayaragavan Muralidharan (St Vincent’s Hospital, Australia) Samantha Phillips, (Northern Hospital, Australia) Wael Jamel, (Box Hill Hospital, Australia) Bung-Kook Ko, Shantanu Joglekar, (Monash Medical Centre, Australia) Ashray Rajagopalan, Joseph Jaya, Yat Cheung Chung, Saania Peeroo, Marek Bak, Jonathan Tiong, (Western Hospital, Australia) Zhei Zhou, (Bendigo Hospital, Australia) Amy Crowe, (Royal Hobart Hospital, Australia) Ryan Newbold, (Launceston General Hospital, Australia) Bethanie Trainor, Mei Lynn Pac Soo, Vaibhavee Khandelwal, (Fiona Stanley Hospital, Australia) Nicholas Eikelboom, Kyungchul Kim, Emily Moran, (Christchurch Hospital, New Zealand) Joshua Hammerschlag, Brendan Desmond, Joel D'Souza, (Middlemore Hospital, New Zealand) Jacky Lu, (Health New Zealand Te Whatu Ora – Southern, New Zealand) Rachel McLay-Barnes, (Auckland City Hospital, New Zealand) Alexandra Gower, (North Shore Hospital, New Zealand) Jenny Choi, (Wellington Regional Hospital, New Zealand) Yu Kai Lim, Douglas Wood, Kate Whytock

**Table S1. Participating sites**

| **Institute** | **Country** |
| --- | --- |
| Austin Hospital | Australia |
| Bankstown/Liverpool Hospital | Australia |
| Bendigo Hospital | Australia |
| Canberra Hospital | Australia |
| Eastern Health | Australia |
| Fiona Stanley Hospital | Australia |
| Flinders Medical Centre | Australia |
| Launceston General Hospital | Australia |
| Monash Medical Centre | Australia |
| Northern Health | Australia |
| Peter MacCallum Cancer Centre | Australia |
| Princess Alexandra Hospital | Australia |
| Royal Brisbane and Women’s Hospital | Australia |
| Royal Hobart Hospital | Australia |
| St Vincent’s Hospital | Australia |
| Auckland City Hospital | New Zealand |
| Christchurch Hospital | New Zealand |
| Dunedin Hospital | New Zealand |
| Middlemore Hospital | New Zealand |
| North Shore Hospital | New Zealand |
| Palmerston North Hospital | New Zealand |
| Wellington Regional Hospital | New Zealand |

**Table S2.** Surgical complication profile according to Clavien-Dindo and ECCG definitions

| **Complications** | **Hybrid**  **MIO**  **(n=61)** | **Total**  **MIO**  **(n=32)** | **OR**  **(95% CI)** | **p-value** |
| --- | --- | --- | --- | --- |
| **Clavien-Dindo complication grade, n (%)** |  |  | - | **0.024** |
| No complications | 18 (29.5) | 17 (53.1) |  |  |
| Grade 1 | 5 (8.2) | 3 (9.4) |  |  |
| Grade 2 | 20 (32.8) | 7 (21.9) |  |  |
| Grade 3 a/b | 16 (26.2) | 4 (12.5) |  |  |
| Grade 4 a/b | 1 (1.6) | 1 (3.1) |  |  |
| Grade 5 | 1 (1.6) | 0 (0.0) |  |  |
| **Pulmonary, n (%)** |  |  |  |  |
| All pulmonary complications | 26 (42.6) | 9 (28.1) | 0.53 (0.22-1.34) | 0.170 |
| Pneumonia | 20 (32.8) | 7 (21.9) | 0.57 (0.23-1.47) | 0.271 |
| Pleural effusion requiring drainage | 9 (14.8) | 3 (9.4) | 0.60 (0.16-2.10) | 0.462 |
| Respiratory failure requiring intubation | 5 (8.2) | 2 (6.3) | 0.75 (0.14-3.88) | 0.735 |
| Pneumothorax requiring intervention | 1 (1.6) | 1 (3.1) | 1.94 (0.10-37.3) | 0.639 |
| Acute respiratory distress syndrome | 2 (3.3) | 1 (3.1) | 0.95 (0.06-8.44) | 0.968 |
| Acute aspiration | 2 (3.3) | 0 (0.0) | - | 0.300 |
| Atelectasis requiring bronchoscopy | 0 (0.0) | 0 (0.0) | - | - |
| Tracheobronchial injury | 0 (0.0) | 0 (0.0) | - | - |
| Air leak >10 days post-surgery | 0 (0.0) | 0 (0.0) | - | - |
| **Cardiac, n (%)** |  |  |  |  |
| All cardiac complications | 4 (6.6) | 1 (3.1) | 0.46 (0.04-3.02) | 0.486 |
| Arrhythmias requiring intervention | 4 (6.6) | 1 (3.1) | 0.46 (0.04-3.02) | 0.486 |
| Congestive heart failure requiring intervention | 1 (1.6) | 0 (0.0) | - | 0.467 |
| Myocardial infarction | 0 (0.0) | 0 (0.0) | - | - |
| Cardiac arrest requiring intervention | 0 (0.0) | 0 (0.0) | - | - |
| Pericarditis requiring treatment | 0 (0.0) | 0 (0.0) | - | - |
| **Gastrointestinal, n (%)** |  |  |  |  |
| Anastomotic leak | 11 (18.0) | 4 (12.5) | 0.65 (0.21-2.17) | 0.491 |
| Conduit necrosis | 3 (4.9) | 0 (0.0) | - | 0.202 |
| Ileus delaying enteral feeding | 1 (1.6) | 2 (6.3) | 4.00 (0.44-58.76) | 0.232 |
| Small bowel obstruction | 1 (1.6) | 0 (0.0) | - | 0.467 |
| Chyle leak | 3 (4.9) | 0 (0.0) | - | 0.202 |
| Pancreatitis | 0 (0.0) | 0 (0.0) | - | - |
| Liver dysfunction | 0 (0.0) | 0 (0.0) | - | - |
| Acute diaphragmatic hernia | 1 (1.6) | 0 (0.0) | - | 0.467 |
| **Infection, n (%)** |  |  |  |  |
| General sepsis | 5 (8.2) | 1 (3.1) | 0.36 (0.03-2.91) | 0.344 |
| Surgical site infection requiring intervention | 2 (3.3) | 0 (0.0) | - | 0.300 |
| Clostridium difficile infection | 1 (1.6) | 0 (0.0) | - | 0.467 |
| Line infection requiring intervention | 1 (1.6) | 0 (0.0) | - | 0.467 |
| Intrathoracic abscess | 4 (6.6) | 2 (6.3) | 0.95 (0.17-4.28) | 0.954 |
| Intraabdominal abscess | 1 (1.6) | 0 (0.0) | - | 0.467 |
| **Neurological, n (%)** |  |  |  |  |
| Delirium | 6 (9.8) | 1 (3.1) | 0.30 (0.02-2.00) | 0.244 |
| Cerebrovascular accident | 0 (0.0) | 0 (0.0) | - | - |
| **Haematological, n (%)** |  |  |  |  |
| Bleeding requiring intervention | 0 (0.0) | 0 (0.0) | - | - |
| Venous thromboembolism | 2 (3.3) | 3 (9.4) | 3.05 (0.59-17.70) | 0.216 |
| **Urological, n (%)** |  |  |  |  |
| Acute kidney injury | 1 (1.6) | 0 (0.0) | - | 0.467 |
| Urinary tract infection | 3 (4.9) | 1 (3.1) | 0.62 (0.04-4.35) | 0.686 |
| **Skin, n (%)** |  |  |  |  |
| Wound dehiscence | 0 (0.0) | 0 (0.0) | - | - |
| Acute abdominal wall hernia | 1 (1.6) | 0 (0.0) | - | 0.467 |

ECCG: Esophagectomy Complication Consensus Guidelines, CI: confidence interval, MIO: Minimally invasive oesophagectomy, OR: Odds ratio.

**Table S3.** Perioperative outcomes for oesophagectomy

| **Perioperative outcomes** | **Hybrid**  **MIO**  **(n=61)** | **Total**  **MIO**  **(n=32)** | **OR**  **(95% CI)** | **p-value** |
| --- | --- | --- | --- | --- |
| Duration of surgery, min, mean (SD) | 465.8 (106.2) | 448.4 (119.4) | - | 0.718 |
| Intraoperative complications, n (%) | 3 (4.9) | 1 (3.1) | 0.62 (0.05-4.35) | 0.686 |
| R1 resection margins achieved, n (%) | 1 (1.6) | 2 (6.3) | 4.00 (0.44-58.76) | 0.232 |
| Lymph node yield, median (IQR) | 19 (15-26) | 20 (17-26) | - | 0.844 |
| Intensive care readmission, n (%) | 8 (13.1) | 3 (9.4) | 0.69 (0.19-2.57) | 0.596 |
| In-hospital mortality, n (%) | 1 (1.6) | 0 (0.0) | - | 0.467 |
| 30-day hospital readmission, n (%) | 13 (21.3) | 2 (6.3) | 0.25 (0.05-1.00) | **0.030** |
| Length of stay, days, median (IQR) | 13 (11-23) | 10 (8-12) | - | 0.567 |
| Textbook outcome achieved, n (%) | 11 (18.0) | 9 (28.1) | 1.78 (0.66-4.55) | 0.260 |
| Commenced adjuvant chemotherapy, n (%) | 42 (68.9) | 26 (81.3) | 1.96 (0.72-5.59) | 0.200 |

CI: confidence interval, IQR: Interquartile range, OR: Odds ratio, SD: Standard deviation.

**Table S4.** Textbook outcomes for oesophagectomy

| **Outcomes** | **Open**  **Oesophagectomy**  **(n=155)** | **Hybrid**  **MIO**  **(n=61)** | **Total**  **MIO**  **(n=32)** | **Open vs Hybrid**  **OR (95% CI),**  **p-value** | **Open vs Total**  **OR (95% CI),**  **p-value** | **Hybrid vs Total**  **OR (95% CI),**  **p-value** |
| --- | --- | --- | --- | --- | --- | --- |
| **Textbook outcome achieved, n (%)** | 27 (17.4) | 11 (18.0) | 9 (28.1) | 1.04 (0.48-2.29) p=0.915 | 1.86 (0.79-4.25) p=0.162 | 1.78 (0.66-4.55) p=0.260 |
| **Textbook outcome criteria components** |  |  |  |  |  |  |
| No major complications, n (%) | 95 (61.3) | 43 (70.5) | 27 (84.4) | 1.51 (0.79-2.83) p=0.205 | **3.41 (1.33-8.48) p=0.013** | 2.26 (0.77-6.04) p=0.140 |
| Hospital stay <14 days, n (%) | 66 (42.6) | 33 (54.1) | 27 (84.4) | 1.59 (0.87-2.94) p=0.126 | **7.28 (2.84-18.01) p<0.001** | **4.58 (1.65-11.93) p=0.004** |
| No hospital readmission, n (%) | 126 (81.3) | 48 (78.7) | 30 (93.8) | 0.85 (0.41-1.71) p=0.664 | 3.45 (0.84-15.31) p=0.084 | **4.06 (1.00-18.91) p=0.030** |
| R0 resection margins achieved, n (%) | 141 (91.0) | 60 (98.4) | 30 (93.8) | 5.96 (0.93-64.24) p=0.054 | 1.49 (0.36-6.87) p=0.609 | 0.25 (0.02-2.25) p=0.232 |
| ≥20 lymph nodes retrieved, n (%) | 91 (58.7) | 30 (49.2) | 16 (50.0) | 0.68 (0.37-1.24) p=0.204 | 0.70 (0.32-1.54) p=0.365 | 1.03 (0.43-2.47) p=0.940 |
| No intraoperative complications, n (%) | 142 (91.6) | 58 (95.1) | 31 (96.9) | 1.77 (0.55-6.00) p=0.381 | 2.84 (0.49-31.18) p=0.303 | 1.60 (0.23-21.43) p=0.686 |
| No anastomotic leakage, n (%) | 124 (80.0) | 50 (82.0) | 28 (87.5) | 1.14 (0.53-2.54) p=0.742 | 1.75 (0.58-4.91) 0.3220 | 1.54 (0.45-4.72) p=0.567 |
| No intensive care readmission, n (%) | 126 (81.3) | 53 (86.9) | 29 (90.6) | 1.53 (0.68-3.35) p=0.326 | 2.23 (0.69-7.32) p=0.202 | 1.46 (0.39-5.38) p=0.596 |
| No in-hospital mortality, n (%) | 152 (98.1) | 60 (98.4) | 32 (100.0) | 1.18 (0.17-15.62) p=0.885 | p=0.428 | p=0.467 |

CI: Confidence interval, MIO: Minimally invasive oesophagectomy, OR: Odds ratio.


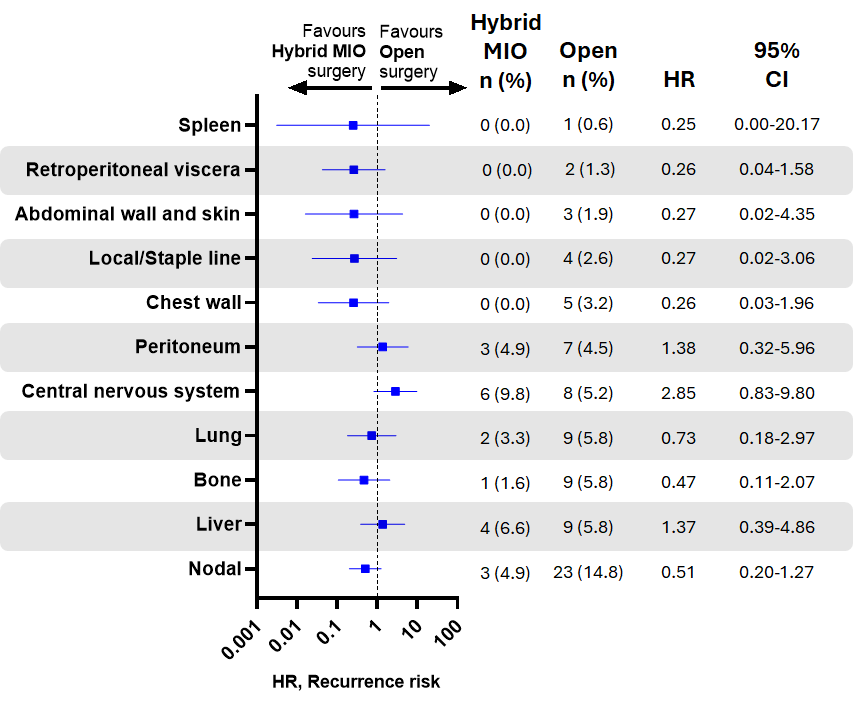


**Figure S1**. Recurrence profile following hybrid minimally invasive versus open transthoracic oesophagectomy across Australian and New Zealand centres. CI: Confidence interval, HR: Hazard ratio, MIO: Minimally invasive oesophagectomy.

**
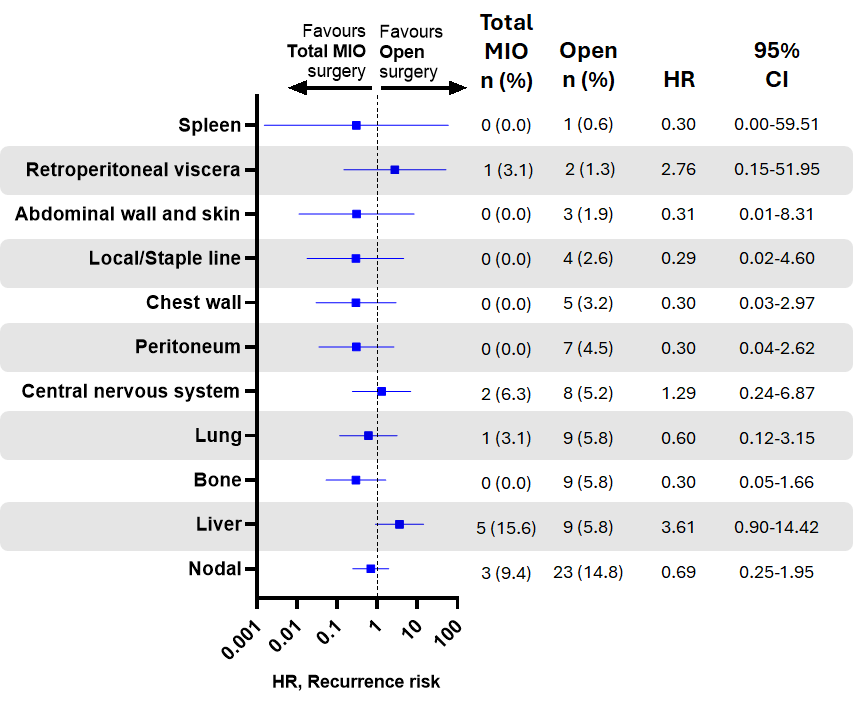
**

**Figure S2**. Recurrence profile following totally minimally invasive versus open transthoracic oesophagectomy across Australian and New Zealand centres. CI: Confidence interval, HR: Hazard ratio, MIO: Minimally invasive oesophagectomy.

**
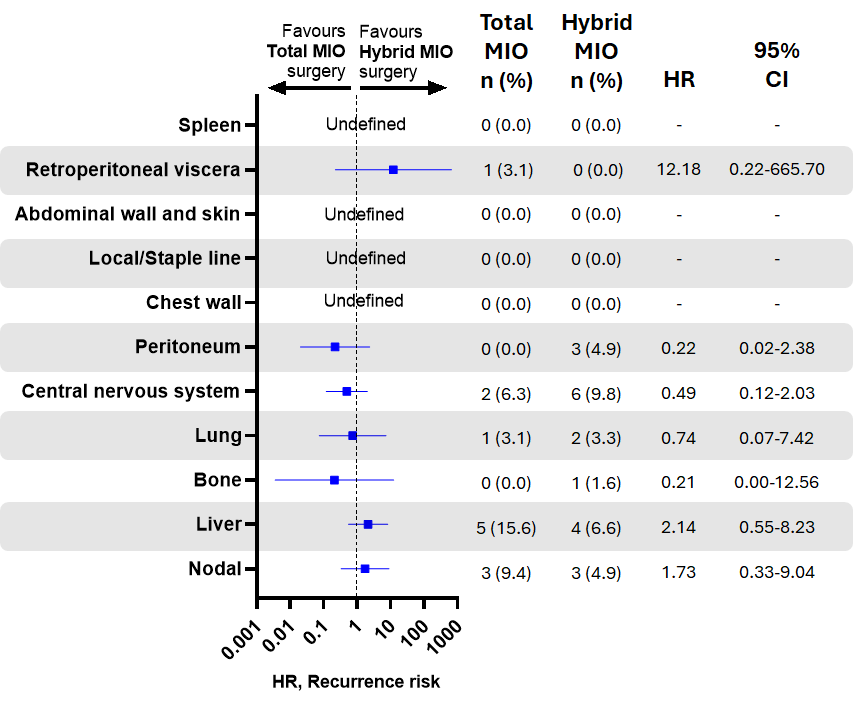
**

**Figure S3**. Recurrence profile following hybrid minimally invasive versus total transthoracic oesophagectomy across Australian and New Zealand centres. CI: Confidence interval, HR: Hazard ratio, MIO: Minimally invasive oesophagectomy.


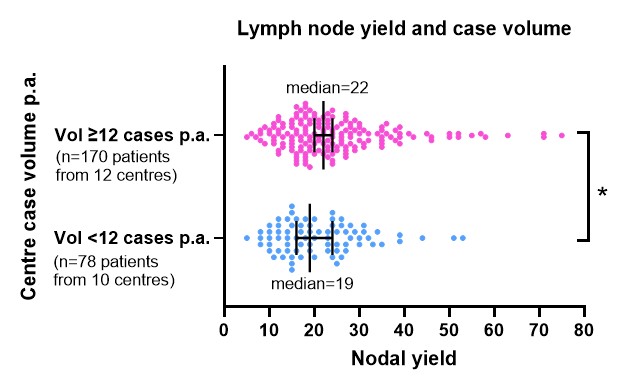


**Figure S4**. Lymph node yield following minimally invasive and open transthoracic oesophagectomy performed across 22 Australian and New Zealand centres. Centre volume is categorised per median oesophagectomy case number per centre per year. *p<0.05.
